# Metabolic Syndrome and Adipokines Profile in Bipolar Depression

**DOI:** 10.3390/nu15214532

**Published:** 2023-10-25

**Authors:** Karolina Bilska, Monika Dmitrzak-Węglarz, Przemysław Osip, Joanna Pawlak, Elżbieta Paszyńska, Agnieszka Permoda-Pachuta

**Affiliations:** 1 Department of Psychiatric Genetics, Poznan University of Medical Sciences, 61-701 Poznan, Poland; 2 HCP Medical Center, 61-485 Poznan, Poland; 3 Department of Integrated Dentistry, Poznan University of Medical Sciences, 61-701 Poznan, Poland; 4 Department of Psychiatry, Medical University of Lublin, 20-059 Lublin, Poland

**Keywords:** metabolic syndrome, bipolar disorder, adipokines, metformin treatment, ADIPO/LEP ratio

## Abstract

Metabolic syndrome (MS) is a growing social, economic, and health problem. MS coexists with nearly half of all patients with affective disorders. This study aimed to evaluate the neurobiological parameters (clinical, anthropometric, biochemical, adipokines levels, and ultrasound of carotid arteries) and their relationship with the development of MS in patients with bipolar disorder. The study group consisted of 70 patients (50 women and 20 men) hospitalized due to episodes of depression in the course of bipolar disorders. The Hamilton Depression Rating Scale was used to assess the severity of the depression symptoms in an acute state of illness and after six weeks of treatment. The serum concentration of adipokines was determined using an ELISA method. The main finding of this study is that the following adipokines correlated with MS in the bipolar depression women group: visfatin, S100B, and leptin had a positive correlation, whereas adiponectin, leptin-receptor, and adiponectin/leptin ratio showed a negative correlation. Moreover, the adiponectin/leptin ratio showed moderate to strong negative correlation with insulin level, BMI, waist circumference, triglyceride level, treatment with metformin, and a positive moderate correlation with HDL. The adiponectin/leptin ratio may be an effective tool to assess MS in depressed female bipolar patients.

## 1. Introduction

Metabolic syndrome (MS) is a growing social, economic, and health problem. MS consists of abdominal obesity, hypertension, and disorders of carbohydrate and lipid metabolism [1]. The prevalence of metabolic syndrome in the Polish population is estimated at about 20%, 18% of men and 22% of women [2]. Insulin resistance and obesity (especially abdominal obesity) plays a key role in the pathogenesis of MS [1,3].

Adipose tissue is a highly specialized tissue that plays an important endocrine function through the synthesis and secretion of adipokines [3,4,5,6]. The enlarged adipocytes secrete pro-inflammatory adipokines, promoting systemic inflammation, and contributing to metabolic syndrome [1]. Among the most important of these adipokines are leptin, resistin, adiponectin, visfatin, and interleukin-6 (IL-6) [1,3,5]. Leptin regulates feeding behavior, energy homeostasis [4,7], and lipid metabolism [5,8,9]. It also controls glucose homeostasis and insulin sensitivity [3,4,9]. Moreover, leptin is known to promote a pro-inflammatory immune response, and it is suggested to be an important factor linking obesity, MS, and cardiovascular diseases [3,8,9]. Another essential role in the development of inflammation is played by resistin [1,5]. The role of resistin in the pathogenesis of insulin resistance in humans remains unclear [4,5]; however, the role in inflammation and metabolic dyslipidemia development is well-known [9]. Adiponectin plays a vital role in the regulation of glucose and lipid metabolism [1,3,5,7]. Adiponectin acts as an endogenous insulin sensitizer, increasing glucose uptake and promoting fatty acid oxidation [7]. Moreover, adiponectin plays a protective role against the development and progression of insulin resistance, MS, and cardiovascular diseases, and also inhibits pro-inflammatory factors [3,7]. Visfatin similarly affects insulin sensitivity due to its insulin-mimetic capacity [1,5,10]. It binds insulin receptors and enhances glucose uptake, transport, and lipogenesis [10]. However, unlike adiponectin, visfatin is a pro-inflammatory mediator that activates multiple inflammatory pathways (e.g., mitogen-activated protein kinase and phosphatidylinositol 3 kinase) [10]. Increased expression of IL-6 has numerous implications for the pathogenesis of obesity and its complications. IL-6, by reducing the expression of the insulin receptor, adiponectin, and inhibiting the activity of lipoprotein lipase, leads to the intensification of insulin resistance [1,11]. Moreover, IL-6, affects the functioning of the vascular endothelium by stimulating the synthesis of acute phase proteins. This leads to the formation and progression of the atherosclerotic lesions inflammation, and dysfunction [1,3]. 

The S100B is a calcium-binding protein responsible for transcriptional regulation and DNA repair, cell differentiation, cell growth and migration, and programmed cell death [12]. The S100B is predominantly expressed by astrocytes [13], and also by other cell types: melanocytes [14], chondrocytes, adipocytes [15,16], skeletal muscle, and a few other cell types [17]. In their research, Fujiya et al. [18] proposed that the S100B functions as an adipokine in the interaction between adipocytes and macrophages. They proved that S100B upregulated the expression of TNF-α and proinflammatory markers in macrophages, and TNF-α augmented S100B secretion from preadipocytes. Moreover, silencing of S100B in preadipocytes significantly reduced TNF-α secretion from macrophages [18]. Recent publications [17,19] suggest the involvement of S100B in obesity and diabetes mechanisms, possibly by participating in the inflammatory processes. 

Bipolar disorder patients are almost twice as likely to have MS than the general population [20,21,22]. Moreover, patients taking antipsychotic medication have a higher risk of developing MS than antipsychotic-free patients [20,21]. Psychiatric patients are more likely to develop obesity and metabolic abnormalities than healthy people [21,22,23]. Factors that predispose psychiatric patients to MS include genetic, unhealthy lifestyle (e.g., smoking, excessive alcohol intake, poor sleep hygiene, physical inactivity, and unhealthy nutritional patterns), but also the use of psychotropic medication (antipsychotics, antidepressants, and mood stabilizers) [20,21,22,23]. 

The aim of this study was to evaluate the neurobiological parameters (clinical, anthropometric, biochemical, adipokines levels, and ultrasound of carotid arteries) and their relationship with the development of MS in patients with bipolar affective disorder. The first research hypothesis is that depending on coexisting MS, patients differ in neurobiological parameters and the level of adipokines tested. The second research hypothesis is that differences in neurobiological parameters and the level of tested adipokines depend on the state of the disease (exacerbation vs. improvement).

## 2. Materials and Methods

### 2.1. Participants

The study group included 50 women (45.0 ± 14.29 years old) and 20 men (50.9 ± 15.73 years old) (Table 1) with a diagnosis of bipolar disorder based on DSM-IV criteria [24,25]. Only patients with current depression episodes were included in the study. Patients were evaluated by psychiatrists twice, upon admission to the hospital in an acute state of illness (exacerbation) and after six weeks of treatment as recommended by the National Consultant [26], to assess depression symptoms using the 17-item version of Hamilton Depression Rating Scale (HAMD) [27]. 

The exclusion criteria were severe and unstable medical conditions, pregnancy, autoimmune diseases, severe and chronic somatic diseases (except diabetes, hypertension, and obesity), infectious diseases four weeks before and during the study period, neuropsychiatric illnesses associated with cognitive impairment, or a prior clinical diagnosis of schizophrenia or schizoaffective disorder. The study protocol did not interfere with the treatment of patients, was compliant with the indications and was supervised by the attending physicians. Patients were treated with various combinations and doses of drugs with different mechanisms of action (antidepressants, antipsychotics, and mood stabilizers). The most often prescribed drugs were: antidepressant—venlafaxine (21 women and 7 men), mood stabilizers—lithium carbonate (23 women and 10 men) and valproic acid (9 women and 11 men), antipsychotics—quetiapine (32 women and 8 men), olanzapine (13 women and 5 men) and clozapine (12 women and 3 men). More detailed information about the pharmacological therapy can be found in the Supplementary Material (Table S1). No patients were in monotherapy or with the same set of drugs in the study group.

Upon admission to the hospital, all patients underwent anthropometric measurements: height, waist circumference (with an accuracy of 1.0 cm), and body weight (with an accuracy of 0.1 kg). These data were used to estimate visceral obesity and body mass index (BMI). Following the recommendations of the World Health Organization (WHO) regarding visceral obesity [28], we adopted the following cut-off points for significantly increased risk of metabolic complications: >88 cm in women and >102 cm in men. BMI below 25 kg/m^2^ was considered a normal body mass index, whereas overweight was diagnosed with BMI values between 25 and 29.9 kg/m^2^, and obesity with a BMI above 30 kg/m^2^ [29]. In the statistical analysis, we distinguished two groups: normal weight and obesity (BMI above 25 kg/m^2^) which consists of overweight, and obesity according to WHO. Biochemical tests determined the lipid profile (HDL, LDL, and TG [mg/dL]) and insulin concentration (mU/mL) were performed. Based on anthropometric measurements and laboratory tests, the internist diagnosed metabolic syndrome under the guidelines of the International Diabetes Federation [30] and decided which patients required metformin treatment (500 mg daily). 

The intima-media complex (IMC) was measured in the common carotid arteries. Distal IMC of both carotid arteries was measured by duplex and B-mode ultrasound using SonoScape S6 ultrasound with a 6–12 Mhz linear transducer. IMC measurements were taken at several points approximately 1 cm proximal to the common carotid sinus. The IMC thickness result is presented as the average of the measurements taken. In addition, the maximum systolic velocity in the tested vessels was determined [31].

The study was approved by the Bioethics Committee of University Medical Sciences in Poznan (Resolution No. 1082/15 of 3 December 2015) [32]. All study participants provided written informed consent and were recruited in 2015–2018 in the Adult Psychiatry Clinic by the Department of Psychiatry, University of Medical Sciences in Poznan.

### 2.2. Biochemical Analysis

The blood samples were collected from the patients twice: upon admission to the hospital and after six weeks of treatment in order to assess the concentration of adipokines in the blood serum. In the exacerbation state, we measured concentrations of visfatin (VIS), adiponectin (ADIPO), S100B, interleukin-6 (IL-6), leptin (LEP), leptin-receptor (LEP_R), and resistin (RES). After six weeks of treatment only ADIPO, VIS, and S100B were measured. Venous blood was collected into EDTA tubes and centrifuged at 1000× *g* for 15 min at 4 °C to obtain serum samples, aliquoted into Eppendorf tubes, and stored at −80 °C. Commercial Enzyme-Linked Immunosorbent Assay tests (ELISA) (Table 2) were used to quantify the selected proteins in the blood serum. Optical density was read with a spectrophotometric plate reader (Asys UVM 340 Microplate Reader from Biochrom Ltd., Cambridge, UK) for a wavelength of 450 nm ± 10 nm. A four-parameter algorithm (four-parameter logistic curve) was used to assay the concentration in the tested samples. All samples and standards were run in duplicates, and the mean value of the two assays was used for statistical evaluation. All ELISA tests were performed according to the manufacturer’s instructions without any modifications.

### 2.3. Statistical Analysis

Statistical analyses were performed using the STATISTICA 13.3 (StatSoft, Krakow, Poland). The significance level *p* < 0.05 was adopted for all analyses. The distribution of variables was studied by the Shapiro–Wilk test. Variables with normal distribution were tested using parametric tests (student’s *t*-test and ANOVA with Tukey’s post-hock test). Variables that did not meet the normal distribution criteria were tested using non-parametric tests (Mann–Whitney U test, Wilcoxon pair order test, ANOVA rank Kruskal–Wallis test, Chi-square test, and Friedman ANOVA with Kendall Concordance). Spearman’s rank correlation coefficient was applied to assess the relationship between the analyzed variables. The ROC curve was used to test the diagnostic ability of the ADIPO/LEP ratio.

## 3. Results

Spearman’s rank correlation coefficient was applied to assess the influence of gender on studied variables. Seven of the examined variables (hypothyroidism, waist circumference, HDL, ADIPO, S100B, LEP, and LEP_R) correlated with sex; therefore, we decided to study women and men groups separately. Depression symptoms upon admission to the hospital in an acute state of illness (exacerbation) and after six weeks of treatment were assessed using the 17-item version of the Hamilton Depression Rating Scale (HAMD) [27]. Hamilton’s total score was higher in exacerbation in both sexes (Table 3). Thirty-five patients (23 women and 12 men) did not achieve remission (score ≤ 7) [33], but they had 25–56% reduction in the total score. A total number of the uptaken antidepressant drugs correlated positively with Hamilton total score (R = 0.3633 *p* = 0.0195). 

Because insulin resistance and obesity play a crucial role in MS pathogenesis, we conducted a statistical analysis of metabolic syndrome, and variables such as insulin levels, BMI, and metformin treatment. Women with MS had higher insulin levels (*p* = 0.0197) and higher BMI values (*p* = 0.007) than women without MS. Unfortunately, these observations were not confirmed in the group of men, probably due to the small study group. We have also tested the thickness and maximum systolic velocity of intima-media complex in the common carotid arteries, but no statistically significant results were obtained in either sex (but were correlated with age, duration of disease, and waist circumference).

Pro-inflammatory adipokines have been tested in the context of obesity, metabolic syndrome, and metformin treatment, as well as concerning the mental state of patients. Obese women had statistically significant elevated levels of S100B and LEP and lower LEP_R levels in exacerbation (Table 4). Women with MS had statistically significant differences in the level of adipokines (VIS, ADIPO, S100B, LEP, and LEP_R) in exacerbations compared with women without MS. The VIS, S100B, and LEP levels have been increased, while ADIPO and LEP_R concentrations have been reduced. Similar results were obtained when comparing three groups: with and without MS, and MS metformin-treated. Post-hock tests showed significant differences not only between patients with and without MS, but also between MS and MS treated with metformin (Table 4). We also compared the ratio of ADIPO/LEP; women without MS (mean ± SD 1.1 ± 0.95) had significantly (Z = 3.90 *p* = 0.0001) higher ratio than women with MS (mean ± SD 0.4 ± 0.76). Moreover, the ADIPO/LEP ratio showed moderate to strong negative correlation with insulin level, BMI, waist circumference, TG level, metformin treatment, and positive moderate correlations with HDL concentrations. We conducted the ROC curve analysis for the ADIPO/LEP ratio regarding MS (Supplementary Table S2). The result was statistically significant (AUC = 0.846 *p* < 0.0001), with the cut-off point at 0.31 (Youden index 0.64). The sensitivity and specificity were 76.5% and 87.5%. In the case of our data, this meant two people without MS were classified as ill, and eight ill people were classified as healthy. We believe that 23.5% of misclassified ill people is too high a percentage, so we decided to set a better-fitting cut-off point. In relation to our data, we have selected a 0.48 cut-off point (Youden index 0.599) with 91.2% sensitivity and 68.8% specificity. In the analysis between the level of adipokines and the mental state of the patients, only the level of visfatin had changed (decreased) significantly (Table 3).

In contrast to women, there were no changes in the level of adipokines in the group of men, in the context of obesity, metabolic syndrome, or metformin treatment (probably due to the small study group). However, the ADIPO/LEP ratio showed a strong negative correlation with BMI and waist circumference (R = −0.6568 *p* = 0.0057 and R = −0.6139 *p* = 0.0088), but no correlation with MS was observed. Interestingly, concerning the mental state of the patients, changes were observed in the level of three adipokines (VIS, ADIPO, S100B), not just one, as was the case in the group of women (Table 3). As in women group, visfatin level decreased while ADIPO and S100B levels increased after six weeks of treatment. 

Spearman’s rank correlation coefficient was applied to assess the influence of neurobiological parameters (clinical, anthropometric, biochemical, adipokines, and ultrasound of carotid arteries) on the development of MS in patients with bipolar affective disorder. In women, thirteen parameters were significantly correlated with MS (Table 5). A positive, strong correlation was observed with metformin treatment, whereas other parameters showed moderate (TG, insulin, BMI, waist circumference, S100B, and LEP) and weak (hypothyroidism and VIS_E) positive correlation (Table 5). A negative, weak correlation was also observed for three parameters: HDL, ADIPO_E, and LEP_R (Table 5). In the men group, only metformin treatment was significantly correlated with MS (R Spearman = 0.8819 *p* < 0.0001). 

## 4. Discussion

In our study, not all patients achieved remission, but they had shown an improvement, defined as a 20–30% reduction in the total scores of HAMD [26]. Bipolar depression is challenging to treat, and drugs should be administrated wisely to avoid mood switches in patients [34,35,36]. Antidepressant therapy is associated with an increased risk of mania or hypomania, so it should be taken with mood stabilizers and/or antipsychotic medications [35]. 

In our study, women suffering from MS had higher insulin levels, BMI, and waist circumference than patients without MS. It is unsurprising because one of the criteria of MS is overweight (mainly abdominal), whereas insulin resistance plays a crucial role in MS pathogenesis [1,3]. BP patients have a higher MS prevalence than the general population [20,21,22]. Most psychiatric patients have at least one metabolic disorder [22,37]. Despite other factors like genetics, physical inactivity, unhealthy diet, and addictions, medications, especially antipsychotic drugs, have well-established weight gain side effects [20,21]. In clinical practice, clozapine and olanzapine are associated with a higher risk of MS [20,21,38,39], quetiapine and risperidone cause moderate alterations [39], whereas aripiprazole has a little effect on body weight [21,38]. Unfortunately, we could not assess the effect of treatment on BMI because patients were weighed only at the time of admission to the hospital. Therefore, it is important to balance the potential benefits and damages in bipolar depression treatment, especially in long-term treatment, because high doses or multiple medications can be associated with harmful metabolic consequences [21,39]. Interestingly, there appears to be a correlation between the higher clinical effectiveness of atypical antipsychotics and the increased risk of metabolic alterations [39]. 

The following adipokines correlated with MS in the women group VIS, S100B, and LEP had a positive correlation, whereas ADIPO, LEP_R, and ADIPO/LEP ratio showed a negative correlation. It has been proven that leptin concentrations are significantly increased in obesity [4,5,40]. The concept of “leptin resistance” was proposed to explain this phenomenon [8]. It assesses that tissues have decreased sensitivity to leptin, so higher leptin levels are essential to correct the metabolic imbalance in obesity [3]. Leptin binds to and activates its transmembrane receptor, the LEP-R, which plays a crucial role in regulating body mass via a negative feedback mechanism between adipose tissue and the hypothalamus [8]. Similarly to our observations, higher leptin level and lowest leptin-receptor concentrations in obese patients were detected in Koch et al. study [41].

Decreased ADIPO levels in patients with obesity [40], coronary heart disease, diabetes, and hypertension demonstrate a high tendency to develop MS [3]. Moreover, patients with BP during the depression episode showed decreased levels of ADIPO [42,43,44]. Obesity and MS are characterized by increased leptin and decreased adiponectin concentration [6,45]. Therefore, the ADIPO/LEP ratio has been suggested as a maker of adipose tissue dysfunction [6,45]. In our study, MS patients reached significantly lower values of ADIPO/LEP ratio than patients without MS, which is consistent with the study conducted by Frühbeck et al. [45]. Moreover, the ADIPO/LEP ratio was negatively correlated with BMI and waist circumference, which is consistent with the previous study [46]. In our study, an insulin level was significantly correlated with the ADIPO/LEP ratio. In the literature, it has been claimed that the ADIPO/LEP ratio correlates with insulin resistance better than adiponectin or leptin alone [6]. In our ROC curve analysis, the statistical software proposed a 0.31 cut-off point with 76.5% sensitivity and 87.5% specificity. The 23.5% of misclassified ill people is too high a percentage, so we have chosen a better-fitting cut-off point 0.48 cut-off point with 91.2% sensitivity and 68.8% specificity. At that point, the specificity is lower, but we believe it is better to order a more detailed diagnostics for a healthy person than to miss an ill person. The ADIPO/LEP ratio has been proposed as a predictive marker for the MS with a cut-off point lower than 0.5 [45].

Visfatin is secreted by adipose and visceral adipose tissue [7], skeletal muscle, liver, and lymphocytes [40]. This cytokine acts like insulin by binding to insulin receptors, increasing glucose uptake [10,47]. The serum visfatin level correlates with the BMI, waist circumference, and insulin resistance index [47]. A meta-analysis study showed a significant increase in visfatin serum concentration in overweight/obese participants compared with normal BMI participants and also in type 2 diabetes mellitus participants compared with the control group [48]. The visfatin serum concentration was also higher in MS patients [49], consistent with our study.

The S100B, which is expressed in adipose tissue [15,16], has been associated with the pathophysiology of obesity-promoting macrophage-based inflammation [17,50]. The serum level of S100B correlates with insulin resistance, metabolic risk score, and fat cell size [17]. In studies conducted on mice [50], plasma and white adipose tissue S100B levels were increased by diet-induced obesity. Also, in a human study, serum levels of S100B positively correlate with BMI; S100B levels in obesity were significantly higher than in overweight and normal weight subjects [51]. In another study, participants with MS had a significantly higher level of S100B than the control group [19]. Moreover, serum levels of S100B were positively correlated with abdominal obesity and triglyceride serum levels [19]. In our study, BP patients with obesity and MS had a higher serum level of S100B in exacerbation than BP patients with normal BMI, and without MS. Our results show, on the one hand, the relationship between the S100B levels and obesity and MS, and on the other hand, with the mental state of patients. The meta-analysis showed elevated levels of serum S100B in patients with affective disorders (depression and mania) compared with the control group [52,53]. The same relationship was also observed in drug-naïve adolescents diagnosed with first-episode unipolar major depression [54].

## 5. Conclusions

We partially confirmed our first research hypothesis: ‘Depending on coexisting MS, patients differ in neurobiological parameters and the level of adipokines tested’. This statement is correct for a women’s group. The second research hypothesis, ‘Differences in neurobiological parameters and the level of tested adipokines depend on the state of the disease (exacerbation vs. improvement)’, is correct both in the groups of women and men but for a different set of proteins.

In this study, we showed that in bipolar depression, adipokines correlated with MS in the women group: VIS, S100B, and LEP had a positive correlation, whereas ADIPO, LEP_R, and ADIPO/LEP ratio showed negative correlation. Moreover, the ADIPO/LEP ratio showed moderate to strong negative correlation with insulin level, BMI, waist circumference, TG level, treatment with metformin, and a positive correlation with HDL correlations. The ADIPO/LEP ratio has been proposed as a predictive marker for MS [6,45], and our study proved that it can be successfully applied in depressed BP women patients. It is necessary to continue research on a larger group with naturalistic treatment, taking into account different patient states: euthymia, hypomania, mania, or mixed states, to check whether the ADIPO/LEP ratio is a good predictor of MS regardless of the patient’s mental state and pharmacological treatment.

## 6. Study Limitations

- Heterogeneous population concerning the treatment used;
- A small group of men and women subgroup;
- Only three adipokines measured twice, in an exacerbation and after six weeks of treatment;
- BMI, waist circumference, HDL, LDL, TG, and insulin level were measured only in an exacerbation state;
- Information on previous treatment was not recorded.

## Figures and Tables

**Table 1:** Characteristic of bipolar patients group with gender distinction.

Anthropometric and Biochemical Parameters	Women Mean ± SD	Men Mean ± SD
Age (years)	45.0 ± 14.29	50.9 ± 15.73
Duration of disease (years)	15.5 ± 8.88	15.9 ± 11.78
HDL (mg/dL)	51.0 ± 15.08	40.1 ± 10.43
LDL (mg/dL)	125.7 ± 39.04	125.3 ± 51.10
TG (mg/dL)	171.6 ± 82.44	196.6 ± 95.25
Insulin (mU/mL)	12.3 ± 10.90	13.3 ± 3.89
BMI (kg/m^2^)	27.7 ± 6.02	28.8 ± 5.80
Waist circumference (cm)	94.9 ± 16.88	103.9 ± 10.35
Comorbidities	Women N (%)	Men N (%)
Metabolic syndrome (MS)	34 (68)	13 (65)
MS metformin treatment	16 (32)	5 (25)
Hypothyroidism	28 (56)	5 (25)
Hypertension	9 (18)	5 (25)
Hypercholesterolemia	4 (8)	1 (5)
Psychiatric drugs	Women N (%)	Men N (%)
Antidepressants	31 (62)	9 (45)
Mood stabilizers	32 (64)	16 (80)
Antipsychotics	39 (78)	12 (60)

Abbreviations: HDL—high-density lipoprotein; LDL—low-density lipoprotein; TG—triglyceride; BMI—body mass index; MS—metabolic syndrome; SD—standard deviation; N- number of patients.

**Table 2:** Parameters of commercial ELISA tests used to assess concentrations of adipokines in the serum.

Adipokine Name	ELISA Kit Name and Manufacturer	Assay Range	Sensitivity	Intra-Assay Variability Coefficient (%)	Inter-Assay Variability Coefficient (%)
Visfatin	E0638h, EIAab, Wucan, China	1.56–100 ng/mL	<0.78 ng/mL	<4.9	<6.4
Adiponectin	DEE009 for the In Vitro Diagnostic (IVD) Demeditec Diagnostics GmbH, Kiel, Germany	0.27–31000 μg/mL	<0.27 μg/mL	<3.7	<8.2
S100B	Human S100B, EZHS100B-33K, EMD Millipore Corporation, Darmstadt, Germany	2.7–2000 pg/mL	2.7 pg/mL	<3.3	<5.4
Interleukin-6	Quantikine ELISA Human Leptin il-6, S6050, R&D Systems Minneapolis, Minneapolis, MN, USA	3.13–100 pg/mL	<0.70 pg/mL	<4.2	<6.4
Leptin	Quantikine ELISA Human Leptin, SLP00, R&D Systems Minneapolis, Minneapolis, MN, USA	15.6–1000 pg/mL	<7.8 pg/mL	<3.3	<5.4
Leptin receptor	Quantikine ELISA Human Leptin sR, DOBR00, R&D Systems Minneapolis, Minneapolis, MN, USA	0.313–20 ng/mL	0.020–0.128 ng/mL	<6.1	<8.6
Resistin	DRSN00 for research use only (RUO) R&D Systems Minneapolis, Minneapolis, MN, USA	0.156–10 ng/mL	0.010–0.055 ng/mL	<5.3	<9.2

**Table 3:** Clinical and biochemical parameters were analyzed at two-time points: upon admission to the hospital in an acute state of illness (exacerbation) and after six weeks of treatment.

Group	Women		Men	
Parameters	Mean ± SD Median (Min–Max)	*p*-Value (t/Z)	Mean ± SD Median (Min–Max)	*p*-Value (t/Z)
HAMD E	16.3 ± 1.84 17 (12–20)	<0.0001 (23.34)	16. 1± 2.19 17 (12–19)	<0.0001 (16.22)
HAMD 6	7.8 ± 2.78 7 (3–15)		8.8 ± 2.48 9 (5–13)	
VIS (ng/mL) E	16.5 ± 7.65 15.9 (2.6–33.4)	<0.0001 (4.10)	16.2 ± 7.95 18.9 (3.7–24.9)	0.0109 (2.58)
VIS (ng/mL) 6	6.4 ± 4.31 5.6 (2.0–18.0)		8.1 ± 5.08 7.4 (1.8–18.8)	
ADIPO (ng/mL) E	7913.3 ± 4840.5 7833.7 (1621.3–24,415.6)	ns	4381.0 ± 2184.65 3899.8 (1912.7–8435.1)	0.0284 (2.19)
ADIPO (ng/mL) 6	9533.5 ± 6994.5 7498.9 (2142.1–27,335.8)		5518.3 ±2900.62 4625.2 (2709.4–10,577.2)	
S100B (ng/mL) E	31.0 ± 18.71 28.25 (4.8–82.6)	ns	29.3 ± 14.52 27.7 (9.1–55.2)	0.038 (2.07)
S100B (ng/mL) 6	27.1 ± 18.94 29.83 (4.7–77.5)		40.2 ± 14.38 40.6 (16.3–66.6)	

Abbreviations: HAMD—Hamilton Depression Rating Scale; VIS—visfatine; ADIPO—adiponectin; E—an exacerbation; 6—after six weeks of treatment; SD—standard deviation; ns—non-significant values. Students’ *t*-test was applied only to HAMD; all other parameters were calculated using the Wilcoxon pair order test, with a significance level of *p* < 0.05.

**Table 4:** The comparison of biochemical parameters concerns three variables: obesity, metabolic syndrome, and metformin treatment in the women group.

Group Parameters	Obesity	Metabolic Syndrome	Metformin Treatment	Metformin Treatment		
				Post-Hock Analysis		
	*p*-Value (Z-Value)	*p*-Value (Z-Value)	*p*-Value (H-Value)	W vs. MSMT *p*-Value (Z-Value)	W vs. MSnT *p*-Value (Z-Value)	MSMT vs. MSnT *p*-Value (Z-Value)
VIS (ng/mL) E	ns	0.0209 (2.31)	0.0360 (6.65)	0.0187 (−2.35)	ns	ns
VIS (ng/mL) 6	ns	ns	ns	ns	ns	ns
ADIPO (ng/mL) E	ns	0.0173 (−2.38)	0.0028 (11.77)	0.0011 (3.26)	ns	0.0143 (−2.45)
ADIPO (ng/mL) 6	ns	ns	ns	0.0428 (2.02)	ns	ns
S100B (ng/mL) E	0.0012 (−3.23)	0.0022 (3.07)	0.0078 (9.71)	0.0122 (−2.51)	0.0055 (−2.78)	ns
S100B (ng/mL) 6	ns	ns	ns	ns	ns	ns
IL 6 (pg/mL)	ns	ns	ns	ns	ns	ns
LEP (pg/mL)	<0.0001 (−4.01)	0.0005 (3.48)	0.0015 (12.97)	0.0006 (−3.41)	0.0083 (−2.64)	ns
LEP_R (ng/mL)	0.0057 (2.76)	0.0254 (−2.24)	ns	ns	ns	ns
RES (ng/mL)	ns	ns	ns	ns	ns	ns

Abbreviations: VIS—visfatin; ADIPO—adiponectin; Il 6—Interleukin 6; LEP—leptin; LEP_R—receptor for leptin; RES- resistin; E—exacerbation; 6—after six weeks of treatment; W—patients without metabolic syndrome; MSMT—patients with metabolic syndrome with metformin treatment; MSnT—patients with metabolic syndrome non treated with metformin; ns—non-significant values; significance level *p* < 0.05. Comparison between normal weight and obesity (Obesity column in the table), patients with and without metabolic syndrome (Metabolic syndrome column in the table), and post-hock analysis were conducted using the Mann–Whitney U test. Comparison between three groups (patients without and with metabolic syndrome, and patients with metabolic syndrome treated with metformin) was conducted using ANOVA rank Kruskal–Wallis test (Metformin treatment column in the table).

**Table 5:** Spearman’s rank correlation coefficient and descriptive statistics for female bipolar patients with and without metabolic syndrome.

Group	Without MS	With MS		
Parameters	Mean ± SD Median (Min–Max)	Mean ± SD Median (Min–Max)	R Spearman	*p*-Value
Age (years)	42.6 ± 15.90 39.5 (21.0–71.0)	46.2 ± 13.57 48.5 (18.0–68.0)	0.1174	ns
Duration of disease (years)	10.9 ± 9.51 10.0 (1.0–35.0)	13.5 ± 8.58 12.0 (1.0–39.0)	0.1661	ns
Hypothyroidism *	6	22	0.3281	0.0362
Hypertension *	2	7	0.083	ns
Hypercholesterolemia *	0	4	0.2254	ns
HDL (mg/dL)	61.2 ± 14.82 60.0 (40.0–81.0)	47.2 ± 13.56 45.0 (24.0–80.0)	−0.4049	0.0129
LDL (mg/Dl)	120.6 ± 32.64 110.0 (92.0–189.0)	141.9 ± 40.62 144.0 (85.0–230.0)	0.2317	ns
TG (mg/dL)	114.2 ± 56.62 106.0 (60.0–250.0)	195.0 ± 80.47 196.0 (59.0–344.0)	0.4657	0.0032
Insulin (mU/mL)	5.45 ± 2.20 5.85 (2.7–7.5)	16.4 ± 12.05 12.6 (3.2–44.6)	0.6161	0.0110
BMI (kg/m^2^)	23.2 ± 3.35 22.5 (19.8–31.2)	29.5 ± 5.96 28.65 (21.2–44.0)	0.5391	0.0003
Waist circumference (cm)	83.5 ± 12.47 79.0 (68.0–108.0)	100.3 ± 16.11 98.0 (72.0–140.0)	0.4882	0.0009
Metformin treatment *	0	16	0.8575	<0.0001
IMC L	0.64 ± 0.26 0.57 (0.43–1.48)	0.65 ± 0.12 0.63 (0.42–0.99)	0.2093	ns
IMC R	0.80 ± 0.88 0.57 (0.35–3.83)	0.65 ± 0.16 0.67 (0.29–1.11)	0.1260	ns
V L	79.9 ± 22.86 77.0 (39.5–116.6)	75.6 ± 14.52 74.1 (52.3–110.5)	−0.0712	ns
V R	84.2 ± 25.71 93.3 (40.7–119.0)	76.1 ± 21.97 78.3 (35.7–127.5)	−0.2185	ns
HAMD E	16.7 ± 1.57 17.0 (14.0–19.0)	16.1 ± 1.95 17.0 (12.0–20.0)	−0.1529	ns
HAMD 6	7.9 ± 2.33 7.0 (5.0–13.0)	7.8 ± 3.00 8.0 (3.0–15.0)	−0.0187	ns
VIS (ng/mL) E	11.9 ± 8.44 11.07 (1.4–31.6)	17.0 ± 6.79 17.3 (2.6–33.4)	0.3350	0.0186
VIS (ng/mL) 6	5.4 ± 4.08 4.5 (2.3–14.3)	6.7 ± 4.34 5.7 (2.0–18.0)	0.2387	ns
ADIPO (ng/mL) E	11657.6 ± 6066.77 9641.0 (3227.1–24,415.6)	7709.9 ± 4782.67 6699.1 (1621.3–23,098.1)	−0.3417	0.0152
ADIPO (ng/mL) 6	12,209.6 ± 7556.85 11,491.7 (5549.0–27,335.8)	8596.9 ± 6734.39 6990.5 (2142.1–25,196.8)	−0.2930	ns
S100B (ng/mL) E	19.9 ± 9.62 17.1 (4.8–36.3)	33.0 ± 15.75 29.67 (13.3–82.8)	0.4398	0.0014
S100B (ng/mL) 6	26.7 ± 13.69 28.2 (4.6–39.5)	30.9 ± 20.67 26.8 (8.9–77.5)	−0.0108	ns
IL 6 (pg/mL)	1.3 ± 0.97 1.2 (0.3–4.2)	2.4 ± 4.55 1.36 (0.1–26.8)	0.1278	ns
LEP (pg/mL)	18,259.1 ± 12,764.8 13,315.5 (3764.0–41,789.0)	41,818.6 ± 24,072.05 37,733.0 (5389.0–93,517.0)	0.4991	0.0002
ADIPO/LEP	1.1 ± 0.95 0.78 (0.09–3.17)	0.4 ± 0.76 0.20 (0.03–4.29)	−0.5586	<0.0001
LEP_R (ng/mL)	33.4 ± 10.04 30.8 (20.7–62.4)	27.3 ± 7.89 26.4 (13.7–47.8)	−0.3209	0.0231
RES (ng/mL)	26.5 ± 10.80 23.0 (11.4–50.7)	28.1 ± 10.65 25.9 (9.6–60.3)	0.0891	ns

Abbreviations: HDL—high-density lipoprotein; LDL—low-density lipoprotein; TG—triglyceride; BMI—body mass index; IMC—intima-media complex; L—left; R—right; V—maximum systolic velocity; HAMD—Hamilton Depression Rating Scale; VIS—visfatin; ADIPO—adiponectin; Il 6—Interleukin 6; LEP—leptin; LEP_R—leptin receptor; RES—resistin; E—exacerbation; 6—after six weeks of treatment; MS—metabolic syndrome; SD—standard deviation; significance level *p* < 0.05; * number of person instead mean ± SD and median (min-max).

## Data Availability

The data that support the findings of this study are available from the second author, MDW, upon reasonable request.

## Supplementary Materials

The following supporting information can be downloaded at: https://www.mdpi.com/article/10.3390/nu15214532/s1, Table S1. List of psychiatric medications (active substances) patients take during six weeks of study; Table S2. The ROC curve analysis of ADIPO/LEP ratio for metabolic syndrome in bipolar depressed women group.
