# Supplementary material for: Metabolic Syndrome and Adipokines Profile in Bipolar Depression
Source: Nutrients. 2023 Oct 25;15(21):4532. doi: 10.3390/nu15214532 (PMC10648184; doi:10.3390/nu15214532)
Supplement: Supplementary file 1 [file nutrients-15-04532-s001.zip › nutrients-2646434-supplementary.pdf]

Table S1. List of psychiatric medications (active substances) patients take during six weeks of study.

	Drugs	Women N (%)	Men N (%)
	**Antidepressants**		
SSRI	Citalpram	1 (2)	0 (0)
	Escitalopram	3 (6)	0 (0)
	Fluoxetine	3 (6)	0 (0)
	Fluvoxamine	1 (2)	0 (0)
	Paroxetine	4 (8)	0 (0)
	Sertaline	7 (14)	2 (10)
SNRI	Venlafaxine	21 (42)	7 (35)
NaSSA	Mirtazapine	9 (18)	5 (25)
	Mianserin	1 (2)	0 (0)
SARI	Trazodone	4 (8)	0 (0)
TCA	Amitriptyline	1 (2)	0 (0)
	Clomipramine	2 (4)	0 (0)
NRI	Reboxetine	4 (8)	0 (0)
NDRI	Bupropion	2 (4)	1 (5)
	**Mood stabilizers**		
	Carbamazepine	2 (4)	3 (15)
	Valproic acid	9 (18)	11 (55)
	Lamotrigine	9 (18)	1 (5)
	Lithuium carbonate	23 (46)	10 (50)
	**Antipsychotics**		
	Aripirazole	4 (8)	0 (0)
	Clozapine	12 (24)	3 (15)
	Quetiapine	32 (64)	8 (40)
	Levomepromazine	0 (0)	1 (5)
	Olanzapine	13 (26)	5 (25)
	Risperidone	0 (0)	2 (10)
	Zuclopenthixol	3 (6)	2 (10)

Abbreviations: SSRI - selective serotonin reuptake inhibitor, SNRI – serotonin-norepinephrine reuptake inhibitor, NaSSA - noradrenergic and specific serotonergic antidepressants, SARI- serotonin antagonist and reuptake inhibitors, TCA - tricyclic antidepressants, NRI - noradrenaline reuptake inhibitors, NDRI - norepinephrine–dopamine reuptake inhibitor

Table S2. The ROC curve analysis of ADIPO/LEP ratio for metabolic syndrome in bipolar depressed women group.

Ratio	MS	W	SV	SP	ACC	PPV	NPV	LR+	LR-	YI
0.029	1	0	0.029	1.000	0.340	1.000	0.327		0.971	0.029
0.040	1	0	0.059	1.000	0.360	1.000	0.333		0.941	0.059
0.046	1	0	0.088	1.000	0.380	1.000	0.340		0.912	0.088
0.052	1	0	0.118	1.000	0.400	1.000	0.348		0.882	0.118
0.060	1	0	0.147	1.000	0.420	1.000	0.359		0.853	0.147
0.070	1	0	0.176	1.000	0.440	1.000	0.364		0.824	0.176
0.071	1	0	0.206	1.000	0.460	1.000	0.372		0.794	0.206
0.076	1	0	0.235	1.000	0.480	1.000	0.381		0.765	0.235
0.089	0	1	0.235	0.938	0.460	0.889	0.366	3.765	0.816	0.173
0.091	1	0	0.265	0.938	0.480	0.900	0.375	4.235	0.784	0.202

0.099	1	0	0.294	0.938	0.500	0.909	0.385	4.706	0.753	0.232
0.124	1	0	0.324	0.938	0.520	0.917	0.395	5.176	0.722	0.261
0.129	1	0	0.353	0.938	0.540	0.923	0.405	5.647	0.690	0.290
0.136	1	0	0.382	0.938	0.560	0.929	0.417	6.118	0.659	0.320
0.138	1	0	0.412	0.938	0.580	0.933	0.429	6.588	0.627	0.349
0.145	1	0	0.441	0.938	0.600	0.938	0.441	7.059	0.596	0.379
0.172	1	0	0.471	0.938	0.620	0.941	0.455	7.529	0.565	0.408
0.189	0	1	0.471	0.875	0.600	0.889	0.438	3.765	0.605	0.346
0.200	1	0	0.500	0.875	0.620	0.895	0.452	4.000	0.571	0.375
0.209	1	0	0.529	0.875	0.640	0.900	0.467	4.235	0.538	0.404
0.223	1	0	0.559	0.875	0.660	0.905	0.483	4.471	0.504	0.434
0.236	1	0	0.588	0.875	0.680	0.909	0.500	4.706	0.471	0.463
0.246	1	0	0.618	0.875	0.700	0.913	0.519	4.941	0.437	0.493
0.248	1	0	0.647	0.875	0.720	0.917	0.538	5.176	0.403	0.522
0.259	1	0	0.706	0.875	0.760	0.923	0.583	5.647	0.336	0.581
0.259	1	0	0.706	0.875	0.760	0.923	0.583	5.647	0.336	0.581
0.281	1	0	0.735	0.875	0.780	0.926	0.609	5.882	0.303	0.610
0.310	1	0	0.765	0.875	0.800	0.929	0.636	6.118	0.269	0.640
0.314	0	1	0.765	0.813	0.780	0.897	0.619	4.078	0.290	0.577
0.323	1	0	0.794	0.813	0.800	0.900	0.650	4.235	0.253	0.607
0.338	0	1	0.794	0.750	0.780	0.871	0.632	3.176	0.275	0.544
0.343	1	0	0.824	0.750	0.800	0.875	0.667	3.294	0.235	0.574
0.358	1	0	0.853	0.750	0.820	0.879	0.706	3.412	0.196	0.603
0.380	1	0	0.882	0.750	0.840	0.882	0.750	3.529	0.157	0.632
0.415	0	1	0.882	0.688	0.820	0.857	0.733	2.824	0.171	0.570
0.484	1	0	0.912	0.688	0.840	0.861	0.786	2.918	0.128	0.599
0.551	0	1	0.912	0.625	0.820	0.838	0.769	2.431	0.141	0.537
0.564	0	1	0.912	0.563	0.800	0.816	0.750	2.084	0.157	0.474
0.695	1	0	0.941	0.563	0.820	0.821	0.818	2.151	0.105	0.504
0.750	0	1	0.941	0.500	0.800	0.800	0.800	1.882	0.118	0.441
0.804	0	1	0.941	0.438	0.780	0.780	0.778	1.673	0.134	0.379
0.964	0	1	0.941	0.375	0.760	0.762	0.750	1.506	0.157	0.316
1.289	0	1	0.941	0.313	0.740	0.744	0.714	1.369	0.188	0.254
1.515	0	1	0.941	0.250	0.720	0.727	0.667	1.255	0.235	0.191
1.776	0	1	0.941	0.188	0.700	0.711	0.600	1.158	0.314	0.129
1.857	1	0	0.971	0.188	0.720	0.717	0.750	1.195	0.157	0.158
2.114	0	1	0.971	0.125	0.700	0.702	0.667	1.109	0.235	0.096
2.876	0	1	0.971	0.063	0.680	0.688	0.500	1.035	0.471	0.033
3.173	0	1	0.971	0.000	0.660	0.673	0.000	0.971		-0.029
4.286	1	0	1.000	0.000	0.680	0.680		1.000		0.000

Abbreviations: Ratio – adiponectin/leptin ratio, MS – patients with metabolic syndrome (1), W – patients without metabolic syndrome (1), SV – sensitivity, SP – specificity, ACC – accuracy, PPV - positive predictive value, NPV - negative predictive value, LR+ - positive likelihood ratio, LR - - negative likelihood ratio, YI - Youden index.
